# Disease Ionomics: Understanding the Role of Ions in Complex Disease

**DOI:** 10.3390/ijms21228646

**Published:** 2020-11-17

**Authors:** Yan Zhang, Yinzhen Xu, Lin Zheng

**Affiliations:** 1 Shenzhen Key Laboratory of Marine Bioresources and Ecology, Brain Disease and Big Data Research Institute, College of Life Sciences and Oceanography, Shenzhen University, Shenzhen 518055, China; xuyinzhen@szu.edu.cn (Y.X.); zhenglin@szu.edu.cn (L.Z.); 2 Shenzhen-Hong Kong Institute of Brain Science-Shenzhen Fundamental Research Institutions, Shenzhen 518055, China; 3 Shenzhen Bay Laboratory, Shenzhen 518055, China

**Keywords:** ionome, ionomics, complex disease, ICP-MS, bioinformatics

## Abstract

Ionomics is a novel multidisciplinary field that uses advanced techniques to investigate the composition and distribution of all minerals and trace elements in a living organism and their variations under diverse physiological and pathological conditions. It involves both high-throughput elemental profiling technologies and bioinformatic methods, providing opportunities to study the molecular mechanism underlying the metabolism, homeostasis, and cross-talk of these elements. While much effort has been made in exploring the ionomic traits relating to plant physiology and nutrition, the use of ionomics in the research of serious diseases is still in progress. In recent years, a number of ionomic studies have been carried out for a variety of complex diseases, which offer theoretical and practical insights into the etiology, early diagnosis, prognosis, and therapy of them. This review aims to give an overview of recent applications of ionomics in the study of complex diseases and discuss the latest advances and future trends in this area. Overall, disease ionomics may provide substantial information for systematic understanding of the properties of the elements and the dynamic network of elements involved in the onset and development of diseases.

## 1. Introduction

Elements are omnipresent and essential for human life. While carbon, hydrogen, oxygen, and nitrogen are the most abundant elements in living systems, many other elements play important roles in maintaining proper structure and/or function of DNA, RNA, and proteins [1]. Except for a small number of elements (called macroelements, such as phosphorus (P), sulfur (S), calcium (Ca), magnesium (Mg), potassium (K), sodium (Na), and chlorine (Cl)), which are used in large quantities for normal functioning, the majority of them belong to trace elements. Biological trace elements (or micronutrients) refer to those dietary elements required in very small amounts (less than 100 ppm) for the development and physiology of an organism; these include iron (Fe), zinc (Zn), copper (Cu), manganese (Mn), molybdenum (Mo), tungsten (W), nickel (Ni), cobalt (Co), chromium (Cr), vanadium (V), selenium (Se), iodine (I), and probably several other elements [2]. Most trace elements are metals and exhibit a wide range of biological functions in enzymatic catalysis, cellular signaling, redox regulation, mitochondrial activity, hormone synthesis and immunological reactions [3,4]. On the other hand, some nonessential elements, such as cadmium (Cd), mercury (Hg), arsenic (As), and lead (Pb), have adverse effects on health, which may cause serious damage [5].

Trace element deficiencies or overload could be directly implicated in various human health problems, which are responsible for a variety of clinical diseases and disorders, such as Fe deficiency in anemia patients and accumulation of Cu and Fe in neurodegenerative diseases [6,7]. Some of these elements may interact and interfere with the bioavailability and essential functions of each other [8]. For example, overuse of Zn supplements may disrupt Cu absorption and lead to neurological problems, and Zn deficiency may result in Fe deficiency anemia and tissue Fe accumulation [9,10]. Moreover, trace element status may be altered in some clinical conditions and could influence the efficacy of the treatment [11]. Therefore, homeostasis of trace elements within the body should be carefully maintained to offer their adequate but not toxic levels for biological processes.

The development of new technologies for capturing and analyzing biological information related to trace elements at different levels has been accelerated in the postgenomic era, which has acquired in-depth knowledge of their utilization and function in humans. Trace element imbalances have been considered as risk factors for many diseases. In the recent decade, with the dramatic increase in omic data (such as genome, transcriptome, and proteome) and a corresponding increase in computational approaches, the complex relationship between trace elements and health or disease has been comprehensively examined. More importantly, the concept of metallome (the complete set of metal ions in an organism) and its extension ionome (all mineral nutrients and trace elements found in an organism) have been introduced [12,13]. The study of the ionome, ionomics, involves quantitative and simultaneous analyses of elemental composition in living systems and changes in this composition under different conditions by using high-throughput elemental analysis technologies and their integration with bioinformatics tools [14]. In the past several years, ionomics has been widely applied in yeast, plants, and most recently mammals (including humans), providing a powerful approach not only to the discovery of new aspects of trace element metabolism and homeostasis but also to our understanding of more complicated networks controlling diverse biological processes that directly/indirectly affect the ionome [15,16,17,18,19]. In addition, elemental speciation and isotopic analyses have also become an inherent or complementary part of ionomics, which are important for an accurate assessment of the function of elements within different biological systems [20,21]. Considering that most of these studies only focus on one or few elements, they need to be extended to a broader range of minerals and trace elements in the future.

Complex diseases, such as diabetes, cancer, and a variety of neurodegenerative and cardiovascular diseases, are thought to be caused by genetic variations (gene–gene interactions) and environmental factors (gene–environment interactions) [22,23,24]. Relationships between trace elements and complex diseases have been discovered for a long time, which raised great interests in exploration of biological function of micronutrients and their potential application for prevention and therapy of these diseases [25,26,27,28]. Previously, numerous studies have been performed to evaluate the status of certain trace elements in physiological and different pathological conditions; however, it is still unclear how disturbed homeostasis of these elements is involved in the development and progression of complex diseases. Until recently, ionomic profiling has been applied to study mineral dynamics for complex diseases (or called disease ionomics), which offers a unique opportunity to explore the mechanisms of these diseases and to search for new diagnostic solutions and therapeutic targets [29,30]. In this review, we briefly introduce the analytical and bioinformatic strategies for ionomic analysis, and then discuss recent advances in the study of ionome for several types of complex diseases.

## 2. An Overview of Ionomics and Its Application in Mammals

**Figure 1:**
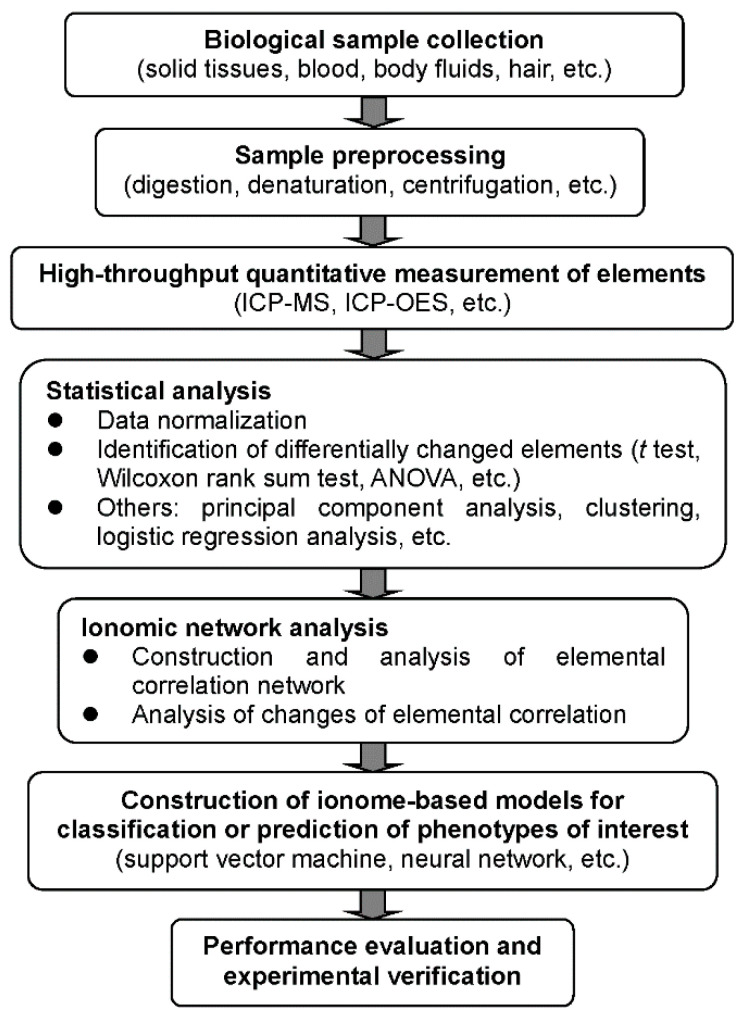
A schematic diagram for the measurement and bioinformatic analysis of the ionome.

In the early days, the study of elemental composition and distribution was mainly used in the fields of ecology and environmental science. The application of ionomics in life science began with plant nutrition research [31]. To date, this approach has been widely used in plants for forward and reverse genetics, screening diversity panels, and modeling of physiological states, which have all been extensively discussed and reviewed in the literature [13,14,18,32,33]. On the other hand, the rapid expansion of ionomics in human health and disease has provided important mechanistic insights into the relationship between trace elements and different diseases, which may aid in the early detection of disease and facilitate the development of new drugs and therapeutic strategies against some of the imbalanced elements. A general procedure for ionome measurement and data analysis is shown in Figure 1.

### 2.1. Ionome Measurement Techniques and Relevant Resources

Accurate determination of multiple elements in various kinds of samples is essential for many areas, such as environmental science, medicine, and agriculture. The majority of experimental techniques for the quantification of elements utilize electronic absorption and fluorescence emission spectra, such as inductively coupled plasma mass spectrometry (ICP-MS), inductively coupled plasma optical emission spectroscopy (ICP-OES), X-ray fluorescence (XRF), and atomic absorption spectrometry [13]. Among them, ICP-MS and ICP-OES are the two most widely used multi-elemental measurement tools due to their outstanding analytical performances. ICP-MS has the ability to detect not only many metals and non-metals at very low concentration (such as part per trillion) but also different isotopes of the same element, whereas ICP-OES offers the advantages of lower cost and simplicity of acquisition and operation [34,35]. In recent years, both ICP-MS and ICP-OES have been successfully used for large-scale ionomic studies in yeast, plants, and animals, which demonstrate the power of ionomics to investigate new aspects of trace element metabolism and status [15,17,18,36,37,38,39,40].

XRF uses characteristic X-rays emitted from an atom that has been excited by the absorption of high energy X-rays or gamma rays for elemental analysis, which is allowing substantial advances in several disciplines of plant science [41]. Recently, synchrotron- and laboratory-based micro-XRF and nano-XRF have been successfully used as a rapid screening and imaging technique for elemental quantification in biological samples, providing a powerful tool to study the spatial distribution of a wide range of biologically relevant elements in various tissues and cells [42,43,44].

With the increasing amount of data generated from various ionomic studies, it is important to develop visual interfaces and management platforms for high-throughput ionomic data acquisition, storage, retrieval, and validation, as well as bioinformatic tools for data analysis. The Purdue Ionomics Information Management System (PiiMS, now called ionomicHUB or iHUB) is the first example of such an integrated workflow system, which allows open access for raw data management, data mining and exploration, and discovery of biomedical knowledge [45]. Currently, this database (https://www.ionomicshub.org/home/PiiMS) contains ionomic resources on more than 240,000 samples, including *Arabidopsis thaliana*, rice, soybean, and *Saccharomyces cerevisiae*, which can be used not only to illustrate the mineral and trace element composition of an organism or tissue but to identify the genes that control the ionome as well. The OPTIMAS Data Warehouse (OPTIMAS-DW) is a comprehensive and integrated database for maize, which contains ionomic and other omic data such as transcriptome, proteome, and metabolome, and allows users to extract data for further analysis and visualization [46]. To date, a disease-related ionomic database is still lacking and urgently needed.

To promote scientific discovery about the ionome and related genes and metabolic pathways, information obtained via experimental or bioinformatic approaches should be correctly annotated and maintained for further utilization. However, systems and tools that allow researchers to annotate new genes involved in trace element metabolism and homeostasis are almost blank. It is clear that, with the rapid increase in the number of novel trace element-related genes and their functional information, new approaches allowing for such efficient and systematized annotation are needed to help bridge the gap between the high-throughput ionomic data and a deep understanding of gene function on a genomic scale.

### 2.2. Bioinformatic Approaches for Ionomic Data Analysis

Bioinformatic methods for ionomic data analysis may include not only basic statistical approaches (e.g., *t* test, Wilcoxon rank sum test, and ANOVA analysis) but also multivariate statistical methods such as principal component analysis, clustering, logistic regression analysis, and discriminant analysis, all of which could also be used for the analysis of other types of omic data [29]. In recent years, systems biology and network-based approaches have been applied to further clarify the complex relationship among different elements and their dynamic changes. Moreover, artificial neural network (ANN) and machine learning algorithms have also been used to build ionome-based classification and prediction models, which may provide important indications for the early detection of various diseases.

### 2.3. Application of Ionomics in Mammals

Previously, a genome-wide siRNA/ionomic screening study was carried out to investigate the mechanisms regulating trace elements in human HeLa cells, which was the first high-throughput ionomic study in mammalian cells [17]. Based on a newly developed computational strategy for ionomic data analysis, a set of novel genes was found to be involved in the metabolism and homeostasis of different trace elements (such as Cu, Se, and Fe), indicating that genes contributing to overall element balance may differ between yeast (or plants) and mammals. Another ionomic study examined 18 elements in multiple organs of 26 mammals, which revealed common and distinct elemental utilization patterns across mammalian organs and species [37]. More interestingly, significant correlations were identified between the ionome and life-history traits, providing insights into the variation of mammalian ionome by organ physiology, lineage specialization, and longevity. For example, liver Zn level was positively linked to body mass, whereas species lifespan correlated positively with liver Zn and Cd and negatively with Se [37].

Very recently, the ionomic profiles of eight organs throughout the lifespan of mice were quantified, which showed new interactions between elements at different levels [40]. The organ ionomes were stable throughout the entire lifespan, and aging was generally characterized by the reduced levels of elements and their increased variance. Based on such a large-scale ionomic dataset, novel organ-specific markers of aging and ionomic clocks were developed to help monitor the aging process and report the effect of calorie restriction, offering a broader view and better understanding of aging from the perspective of elemental composition.

## 3. Ionomics of Complex Disease

Over the past several decades, a great number of studies have revealed that imbalance of trace elements may be one of the risk factors of many complex diseases; however, the underlying molecular mechanisms and the concentration–effect relationship have not been well clarified. Before the concept of ionomics was developed, ICP-MS had been used to measure the concentrations of multiple trace elements (especially metals) in body fluids, hair and nails, and other disease-related tissues [47]. Compared with biofluids, hair and nail samples have demonstrated long-term stability of nutritional status, which often serve as important indicators for monitoring mineral content and toxic metal accumulation in various populations [48,49]. It should also be noted that they are prone to be affected by either the application of cosmetic treatments (such as dyeing and bleaching) or external metal contamination and that they may be more fit for population groups than single individuals [48,49,50]. It was suggested more than thirty years ago that ICP-MS could be applied to examine the levels of multiple elements in brain tissue, cerebrospinal fluid (CSF), and serum from Alzheimer’s disease (AD) victims and matched control subjects [51]. Similar preliminary research studies were also conducted for some other diseases such as diabetes, coronary heart disease, Parkinson’s disease (PD), autism, viral infection, and several types of cancer [52,53,54,55,56]. Most of these studies only focused on simple analysis and description of single or a limited number of elements. Although some disease-related elements have been reported, interactions between elements and disease-specific elemental networks are not yet clear. With the fast development of bioinformatics and systems biology over recent years, advanced computational methodologies (such as network-based methods and machine learning algorithms) have been more and more frequently employed for systematic analysis of ionomic data. Depicting the complex inter-relationship between elements is one of the major purposes of current disease ionomics, which may not only improve our understanding of the roles of individual elements and their cross-talks in the pathogenesis and progression of complex diseases but also help to discover new signatures for early disease detection and curative effect evaluation. As most ionomic results to date have been derived from cross-sectional or case-control studies, the correlation between elements and disease may not implicate a causative relationship, which should be examined via extensive intervention or longitudinal studies [57,58]. In addition, the intrinsic limitation of current elemental analytical techniques and the reliability and validity of using ionomic information as new markers of different diseases imply that there is still a long way to go before ionomics can be more widely used in clinics. However, recent application of ionomics in complex diseases has provided compelling evidence for the potential benefits of disease ionomics, which reveals strong relationships between trace elements and the onset and progression of different diseases and may help improve early diagnosis and therapy of them in the future. Table 1 shows a summary of the major results of published ionomic studies for a variety of complex diseases. In the following sections, we will focus on several types of complex diseases and discuss recent progress on ionomic studies for them.

### 3.1. Diabetes

Diabetes mellitus is a group of metabolic diseases characterized by chronic hyperglycemia, which may result in long-term damage, dysfunction, and failure of various organs [105]. It comprises several types, such as type 1 diabetes (T1D), type 2 diabetes (T2D), and gestational diabetes (GD). Among them, T2D accounts for about 90% of all the cases worldwide and is caused by the disruption of the insulin signaling pathway (insulin resistance). The number of patients with T2D has rapidly and continuously increased in recent years, which has become a serious global public health problem. Emerging evidence has suggested that ion homeostasis may play important roles in T2D and some other related metabolic abnormalities [106].

Skalnaya et al. examined hair trace element contents in Russian women with obesity and T2D and found that significantly elevated K, Na, Hg and decreased Ca, Mg, Zn, Co levels were associated with T2D [59]. In addition, highly similar changes in hair elemental status were detected in both obese and diabetic women when compared to control subjects, suggesting a general pathophysiological mechanism underlying metabolic mineral disturbances. Flores et al. analyzed the concentrations of at least 14 elements in serum and urine samples of 76 patients with T2D and observed a possible antagonistic interaction between Mo and Cu, which might be involved in the progression of diabetic complications [54]. Sun et al. measured the fasting plasma elemental concentrations to investigate associations of ion modules/networks with obesity, metabolic syndrome and T2D in approximately 1000 middle-aged Chinese men and women [60]. Based on the advanced mutual information approach, they showed that Cu and P always ranked the first two among the specific ion networks related to the above metabolic abnormalities, implicating potential associations of the two elements with T2D and other metabolic disorders. Liu et al. conducted a population-based study to analyze urine ionome of more than 2100 Chinese aged 55 to 76 years, which revealed that increased urinary Ni concentration is associated with elevated prevalence of T2D, indicating the potential harmfulness of Ni exposure in the pathogenesis of T2D [61]. Another Chinese population-based case-control study explored the associations between the prevalence risk of T2D and plasma levels of 20 trace elements and heavy metals, and found that several heavy metals (such as Mn, Cu, Zn, As, and Cd) were positively associated with the morbidity of diabetes, implying a significant impact of environmental metal levels on the risk of developing T2D [62]. Badran et al. applied multivariate statistical techniques to assess the levels of 24 trace elements in the serums of T2D patients in Egypt, which suggested that Mg, Fe, Cu, and Zn appeared to be the most crucial factors associated with T2D [63]. Very recently, Marín-Martínez et al. analyzed the saliva and plasma levels of 19 trace elements in patients with T2D and their association to metabolic control and the presence of chronic complications [64]. Decreased Co level in saliva and increased strontium (Sr) level in plasma were observed in the diabetic patients with chronic complications, providing complementary information for predicting diabetic complications. Another study examined the relationship between microvascular complications of T2D and blood trace element levels in Turkey, which showed a potential association between low Mg and Cr levels and microvascular complications in patients with T2D [65]. Overall, it seems that diabetic patient populations from different regions/ethnicities and different body fluids/tissues from same patients have specific elemental profiles, which demonstrates high complexity and tissue specificity of the ionome.

In addition, same body fluids (say, blood) from the same patient populations may also have somewhat different ionomic patterns at different stages of diabetes. Recently, one research group from Norway carried out two separate ionomic studies for T2D [66,107]. In one case-control study, they investigated the association between blood ionome and the prevalence of early phase of T2D (previously undiagnosed, screening-detected T2D) [66]. Using multivariable conditional logistic regression models, they showed that increased levels of several metals (Cd, Cr, Fe, Ni, Ag, and Zn) and decreased levels of bromine (Br) are associated with increased risk of T2D, implying a possible role of these metals in the development of T2D. In the other parallel study, they analyzed the relationship between T2D duration and blood levels of trace elements related to T2D prevalence and found that Ca levels are positively associated with diabetes duration, suggesting that Ca may be linked to the progression of T2D [107]. Unfortunately, so far ionomic study has not been performed in pre-diabetes that precedes the onset of T2D and is characterized by impaired fasting glucose and impaired glucose tolerance. Investigation of the ionomic profiles in pre-diabetes patients should be helpful to comprehensively understand the alternations of trace element levels during the progression of pre-diabetes to T2D [108].

Ionomic studies have also been reported for several other types of diabetes. Forte et al. analyzed the blood levels of multiple trace elements in subjects with T1D and T2D living in Sardinia (an Italian insular region with a high incidence of T1D), which revealed that T1D was associated with Cr, Mn, Ni, Pb, and Zn deficiency while T2D with Cr, Mn, and Ni deficiency when compared to controls, and that Pb was the only metal that was significantly different between the two diabetic types (higher in T2D) [67]. Peruzzu et al. further evaluated the correlations between these elements and lipid profiles and glycaemic control in patients with T1D from the same region, and found that Zn, Fe, and Se were correlated with different kinds of lipids, while Cr and Cu were significantly correlated with fasting plasma glucose and glycated haemoglobin levels in males, respectively [109]. Elevated serum levels of Cu and Mo, as well as lower levels of Mn, Zn, and Se, were also reported in subjects with T1D in the U.S., suggesting that an alteration in the balance of these essential elements may be an important hallmark of the disease [68]. For the first time, Roverso et al. analyzed the ionomes of the placentas from women affected by GD and healthy women, and showed a decreased level of Cd and increased level of Se in GD placentas, implying that they are two key elements for understanding the molecular pathways of GD [69]. Very recently, they examined the ionomes of placenta, maternal whole blood, and umbilical cord blood samples from more GD and control pregnant subjects [70]. Interestingly, many elements detected in the umbilical cord blood of fetuses were found to be significantly correlated with GD, which may give us the possibility to use cord blood for helping to understand the biochemical processes occurring during GD and to delineate more accurate nutritional guidelines for pregnant women. Further effort is needed to investigate the molecular mechanisms underlying mineral nutrient dyshomeostasis in patients with different types of diabetes.

### 3.2. Neurodegenerative Diseases

Neurodegenerative diseases represent a group of chronic progressive diseases or disorders that mainly occur in old age and are characterized by the functional deterioration and ultimate loss of neuronal cells in specific brain regions [110]. The major neurodegenerative diseases include AD, PD, Huntington’s disease (HD), and amyotrophic lateral sclerosis (ALS). Previous epidemiological and experimental studies have shown a significant relationship between trace element dyshomeostasis and the onset and progression of neurodegenerative diseases [111,112,113,114]. In the recent decade, ionomics or metallomics has been applied to the study of these diseases, which may help to understand the biochemical changes and to discover new drug targets of these illnesses [115].

AD is a socially significant chronic neurodegenerative disease characterized by irreversible and progressive impairment of memory and cognitive functions, and is identified as the most common cause of dementia [116,117]. The neuropathological hallmarks of AD include the accumulation of beta-amyloid (Aβ) plaques and neurofibrillary tangles (composed of abnormally hyperphosphorylated tau) as well as the loss of neurons and synapses in the brain [118]. Although the pathogenesis of AD is still unclear, it is well known that metal ions play an important role in both Aβ deposition in senile plaques and tau phosphorylation [119,120]. Gerhardsson et al. measured the concentrations of 19 elements in both plasma and CSF in patients with AD, patients with the combination of AD and minor vascular components (AD + vasc), and healthy controls [71]. Compared to controls, elevated concentrations of Mn and Hg in plasma and decreased concentrations of V, Mn, Pb, and several other trace metals in CSF were observed in subjects with AD and AD + vasc; however, no consistent metal pattern in plasma or CSF could be detected in patients with AD, suggesting specificity of ionomic profiles in different body fluids. Moreover, they also reported different relationships between the concentrations of some of these metals and the levels of well-known AD markers including Aβ, total tau (T-tau), and phosphorylated-tau (P-tau) in CSF, such as positive correlations between T-tau and P-tau and Mn as well as negative correlation between T-tau and cesium (Cs) [121]. To study the progression of dementia, González-Domínguez et al. analyzed the serum ionomes of healthy people, AD and mild cognitive impairment (MCI) patients [72]. Some metals, such as Fe, Cu, Zn, and aluminium (Al), appeared to have progressive changes with the development of neurodegeneration, whereas some other elements (e.g., Mn and V) were only found to have a strong relationship with early neurodegenerative changes, indicating that these metal abnormalities can be related to different biological processes (such as oxidative stress, altered metal homeostasis, and impaired glucose metabolism) involved in the decline of cognitive functions. Recently, Xu et al. performed a case-control study of multiple elements in the plasma of AD patients and matched controls [122]. Although no differential element could be detected, several co-regulated metal pairs were found to be associated with AD, which could be used as potential peripheral indicators of AD status [122]. In addition to blood- and CSF-based studies, ionomic profiles of other tissues from AD patients were also examined. For example, Koseoglu et al. measured the hair and nail concentrations of a variety of trace elements in AD patients at different clinical stages, which revealed significant tissue-specific differences between the patient and healthy control groups [73]. Interestingly, alkali metals were found to be correlated with the severity of AD, especially nail Na level, suggesting that the accumulation of these metals might be important in the progression of this disease.

Although AD mainly affects the structure and function of the brain and many previous studies have shown a close relationship between metal dyshomeostasis in different brain regions and the development of AD [123,124,125], ionomic studies based on abnormal brain tissues relevant to AD are very rare and have only been conducted in animal models. For example, Ciavardelli et al. evaluated the levels of multiple elements in brains and cerebella of the triple-transgenic AD (3xTg-AD) mice and identified significant changes in elemental profiles in 3xTg-AD mice when compared to the wild-type mice [126]. They also analyzed ionomic variations in the mouse brain after a long-term dietary supplementation of Zn. Zheng et al. performed a multi-time-point ionomic analysis to investigate the interactions among 15 elements in the brain by using 3xTg-AD mice receiving high-dose sodium selenate dietary supplementation, which revealed that Se intake could significantly reduce the concentrations of a variety of elements in the brain, especially Fe (a known risk factor for AD) whose level was completely reversed to normal state [127]. In addition, by building the elemental correlation network, significant and time-specific elemental correlations and correlation changes were identified. Such a highly complex and dynamic cross-talk between Se and other elements may provide new mechanistic clues concerning the potential use of selenate for ameliorating AD-related symptoms and pathology [127,128].

PD is the second most common neurodegenerative disorder predominantly affecting dopaminergic neurons in the substantia nigra of the brain stem, which is clinically characterized by a series of motor impairments including tremor, rigidity, bradykinesia, and postural instability and can be divided into different subtypes [129,130]. Several early studies have been carried out to examine the relationship between PD and trace elements and to search for new markers for PD pathology or progression in biological fluids based on relatively small patient cohorts. Forte et al. quantified the levels of multiple metals in urine, serum, blood, and CSF of patients affected by PD and age-matched controls, which demonstrated that each of the examined body fluids has significantly changed metals, such as a decreasing trend for Al in all the fluids and an elevated level of Ca in urine, serum, and blood of PD patients [74]. Hegde et al. reported a dynamic disturbance in element homeostasis as well as inter-element relations in the serum during the progression of PD [75]. Alimonti et al. assessed the concentrations of 26 elements in the CSF of 42 patients with PD, which suggested that significantly lower levels of Pb, Cr, and Fe might be the most suitable elements to distinguish between normality and PD condition [53]. However, bias may have existed in those results due to the limited number of patients. In recent years, large-scale ionomic studies have been performed to provide more reliable evidence for the interaction between trace elements and the development of PD. For example, Zhao et al. measured the levels of several trace elements in plasma from 238 PD patients and 302 controls recruited from eastern China, and found that lower plasma Se and Fe levels may reduce the risk for PD, whereas lower plasma Zn might be a PD risk factor [76]. Based on the plasma concentrations of these trace elements and other clinical features, a new model for prediction of PD patients was proposed, which achieved an accuracy of over 80%. Sanyal et al. integrated ionomics, multivariate and ANN approaches to study elemental profile variations in CSF and serum of PD patients from a large cohort of Indian population [77]. Several elements such as Ca, Mg, and Fe were found to be significantly changed in both fluids. Moreover, a neural network-based classifier was developed based on differentially changed elements, which provides 99% accuracy in PD prediction from CSF and serum. Besides body fluids, hair elemental profiles were also analyzed in relatively small cohorts of PD patients, which showed contradictory results [131,132]. Althouth previous studies have reported a strong relationship between Mn exposure and PD [133,134], most of the above studies did not show significant difference in total Mn level between PD patients and controls. However, recent speciation analysis of Mn in serum revealed that the level of Mn–albumin complexes in PD patients was much higher than in controls, implying a redistribution of Mn between high and low molecular weight ligands [132]. Similar to AD, brain tissue-based ionomic studies have only been performed in animal models. For example, increased concentrations of Fe, Mn, and Cu in the lesioned substantia nigra were previously observed in the 6-hydroxydopamine-induced PD mouse model, indicating a potential role of these essential metals in the pathogenesis of PD [135]. However, variations in the brain ionome of PD patients are not yet clear.

Besides AD and PD, ionomic studies have also been conducted for several other neurodegenerative diseases such as HD and ALS. HD is an autosomal dominant neurodegenerative disorder resulting in progressive motor, psychiatric, and cognitive impairment, and dyshomeostasis of certain metals in the brain may contribute to neuropathogenesis of HD [136,137]. Very recently, Squadrone et al. reported abnormal concentrations of several trace metals in the blood of HD patients, suggesting that the use of blood metal profile may help identify potential metal targets for a novel therapeutic approach against HD [78]. ALS is an adult-onset progressive and fatal degenerative disorder of motor neurons with unknown etiology. In the last decade, metal dysregulation has been proposed to be involved in ALS pathophysiology [138]. Roos et al. analyzed metal concentrations in CSF and plasma in a small cohort of ALS patients and found that levels of several neurotoxic metals (such as Al, Cd, and Cu) were significantly increased in CSF but not in blood plasma of ALS patients, implying that accumulation of these toxic metals in the brain has an impact on the pathogenesis of ALS [79]. Another study analyzed the concentrations of a broad spectrum of metals in serum and whole blood of sporadic ALS patients and found that higher concentrations of Se, Mn, and Al and lower serum concentration of As were associated with this disease [80]. In order to investigate the possible role of trace elements in the severity of ALS, Oggiano et al. analyzed the content of a variety of essential and heavy metals in blood, urine, and hair of ALS patients with different stages, which suggests a protective role of Se and a risk factor in the presence of Pb in blood [81]. A major limitation of these studies is the relatively small number of patients analyzed, which could restrict the power of ionomic evaluation especially considering the high variability of trace element contents in biological fluids [139]. Nevertheless, ionomics may be quite useful to gain a comprehensive view of the complex interactions between trace elements and neurodegenerative diseases.

### 3.3. Cancer

Cancer is one of the most common diseases that cause a high death rate. The development of cancer is a very complex process in which metals have been found to be critically involved. Lots of studies have been carried out to investigate the relationship between one or a few metals and different cancers, such as the association between dietary intake of metals and risk of cancer, possible carcinogenic mechanisms of metals, and epidemiological evidence linking prolonged heavy metal exposure to cancer occurrence [140,141,142,143]. In recent years, ionomic approaches have been used to systematically examine the variations of elemental profiles in different body fluids and tissues (including cancerous tissues) for several types of cancer (such as prostate, lung, pancreatic, and gastrointestinal cancers), which not only provide valuable information to better understand the complex interactions between elements and cancer, but also offer considerable potential of ionomic studies for cancer biomedical purposes.

Prostate cancer (PC) is the most prevalent cancer in men with a high mortality rate, and exposure to toxic metals is one of the most important factors in the aetiology of PC. An early study measured the concentrations of 20 trace elements in the hair samples collected from patients with PC and healthy people. Based on a support vector machine algorithm, a prediction model for PC with the prediction accuracy of 95% was developed, which might be used to predict the risk of PC in the clinics [82]. Another study analyzed the elemental profiles in the blood, scalp hair, and nails of PC patients and found that increased concentrations of Fe, Cd, Mn, Ni, and Pb and decreased concentration of Zn were present in all or almost all examined tissues of the patients compared with healthy controls [83]. Moreover, divergent ionomic variations were also observed for different types and stages of PC, suggesting presumptive benefits of these elements in the diagnosis/prognosis of PC. In recent years, somewhat different variations of trace element contents were also reported in different cohorts of patients. Karimi et al. reported that low levels of Se and Zn and high levels of Cu, Fe, and Mn appeared to be associated with the risk of PC [84]. Lim et al. analyzed the correlation between serum concentrations of multiple heavy metals and PC risk, which revealed positive associations between the serum levels of As and Zn and PC risk [85]. Very recently, Saleh et al. suggested that decreased levels of Se, Zn, and Mn and increased Cu and Fe levels in the serum may play significant roles in the initiation of PC [86]. Considering that these studies involved only a limited number of patients (mostly less than 100 subjects), future research with much larger patient cohorts (including different PC subtypes and stages) is clearly needed.

Lung cancer (LC) is one of the leading causes of cancer-related death worldwide, and it is known that trace elements play important roles in the carcinogenic process of LC. One early study analyzed the elemental levels of the scalp hair from patients with non-small cell lung cancer (NSCLC) and healthy controls, which revealed a strong correlation between the carcinogenic processes of NSCLC and trace elements, especially heavy metals whose accumulation in the body may pose a high risk for LC development [87]. Lee et al. examined the concentrations of 14 elements in the pleural effusion of patients with LC and found that only the concentration of Zn was significantly lower in all smokers than in non-smokers, implying that cigarette smoke may contribute to Zn deficiency in the pleural effusion of smokers with LC [144]. Callejón-Leblic et al. conducted a cross-sectional study to analyze essential and toxic elements in serum, urine and for the first time in bronchoalveolar lavage fluid (BALF) samples from LC patients, which demonstrated that several metals (such as V, Cr, and Cu in serum, Cd in urine, and Mn in BALF) and metal ratios/correlations could be used as important markers of LC in different body fluids [88]. Very recently, Qayyum et al. also reported significantly increased levels of several heavy metals (such as Cr, Cd, Pb, Hg, As, and Ni) in the serum of female patients with LC from Pakistan, suggesting that accumulation of toxic metals may increase the risk of LC in female populations [89]. Some other studies that only examined the levels of specific elements in different body fluids and tumor tissues also reported statistically significant differences from the distribution of certain elements, such as decreased Zn and elevated Cu concentrations in serum, and decreased Fe and Mn levels in urine and tumor tissues of LC patients [145,146,147]. Based on previously published metallomic/ionomic results, a recent review discussed the critical role of metals in LC, especially the importance of high exposure of metals, metal homeostasis and interactions, and metal speciation in cancer onset and progression, which indicates the potential use of such information in early diagnosis, prognosis, or therapy for LC [148].

Pancreatic cancer (PaC) is one of the most fatal and aggressive malignancy cancers with poor prognosis and survival, whose early diagnosis is very difficult. Amaral et al. used ionomic approaches to evaluate the association between concentrations of trace elements in toenails and PaC risk [90]. Elevated concentrations of Cd, As, and Pb could increase the risk of PaC whereas high levels of Se and Ni were inversely associated with PaC, indicating an important role of these elements in pancreatic carcinogenesis. Camargo et al. also reported that PaC patients occupationally exposed to certain chemical agents or mixtures (e.g., aromatic hydrocarbon solvents and pesticides) had higher concentrations of heavy metals such as Cd, Pb, V, Mn, and As in toenails, suggesting that these elements may account for some of the occupational risks for PaC [149]. Two recent ionomic studies investigated the complex relationship among trace elements in toenails, KRAS mutations, and survival in pancreatic ductal adenocarcinoma (PDAC, the most common form of PaC), which not only suggested a special role for several trace elements (such as Ni and Mn) in pancreatic and perhaps other cancers with KRAS mutations, but also found a strong association between higher toenail concentrations of heavy metals (such as Pb, Cd, As, and V) and better survival of PDAC [150,151]. In addition, a very recent urine ionomic study of PDAC showed remarkable differences for several essential metals (including significantly lower levels of Ca and Mg and higher levels of Cu and Zn in PDAC), implying that ionomics is a promising approach for discovery of early diagnostic markers for PDAC [91]. Similar variations have previously been reported in the studies of corresponding metals in serum of PDAC patients [152,153]. Unfortunately, an ionomic analysis in the blood samples of PaC is still lacking.

Ionomic studies have also been reported for several types of gastrointestinal cancer, including colorectal, gastric, and esophageal cancers. Considering that the variations in the distribution of trace elements in cancerous and normal tissues may play a role in the development of cancers, a few ionomic studies have tried to address such differences in patients with colorectal cancer (CRC). An early ionomic study analyzed 18 elements in tumorous and adjacent non-tumorous paired samples from patients with CRC in Spain and found that the accumulation of a number of essential elements (such as Mn, Se, Cu, and Fe) could be used as an indicator of cancer progression [92]. A recent Asian cross-sectional study also evaluated trace element and heavy metal levels in cancerous and non-cancerous tissues of CRC patients, which showed somewhat different changes of elemental profiles and suggested that additional metals (such as increased Zn and Cr and decreased Mn levels) may play a role in developing CRC [93]. Kohzadi et al. compared the concentrations of trace elements in gastric cancer tissues and normal tissues and found higher levels of Fe, Mg, and As and lower levels of Cr, Cu, Ca, and Ni in cancerous tissues compared with non-cancerous tissues [94]. Lin et al. used ionomic and machine learning methods (such as random forest and support vector machine algorithms) to investigate the association between the alterations of serum elemental profiles and esophageal squamous cell carcinoma (ESCC), which suggested the potential promising application of ionome in diagnosis and prognosis of ESCC [95]. Very recently, based on the comparative evaluation of elemental concentrations in cancerous and non-cancerous esophageal tissues from the same patients with ESCC, Xie et al. identified significantly changed elements and established a diagnostic model of ESCC, which showed excellent performance in the classification of cancerous and non-cancerous group [96].

Besides the above cancer types, ionomics has also been applied for several other cancers. Golasik et al. analyzed the status of multiple essential and toxic elements in hair and nails from patients with larynx cancer and healthy controls, which revealed that the levels of Zn and Fe were significantly decreased in the patient group whereas heavy metals were observed to have the opposite trend [97]. Moreover, using elemental analysis and other information, several statistical models based on ANN, canonical discriminant analysis, and logistic regression were also built for the prediction of cancer probability, which may be used as a useful tool for risk estimation and early screening of laryngeal cancer. Burton et al. examined urinary ionome in women newly diagnosed with breast cancer and found that Cu and Pb were significantly increased in the patients [98]. In addition, a multivariate model comprising Cu, Pb, and patient age information was constructed for the early detection of breast cancer. Very recently, Lee et al. examined the associations of a variety of metals in serum with gallbladder cancer (GBC) and with gallstones (an important risk factor for GBC), which provides cross-sectional evidence for a strong correlation between temporality of metal exposure and the natural history and mechanisms of GBC [99].

It should be noted that current development of cancer ionomics is still in the primary stage as it has only been used for a limited number of cancer types; however, one of the greatest benefits of this approach is to depict the cross-talk between elements (including both essential and harmful elements) and cancer pathogenesis to further understand the risk factors and determinants from health status to disease. In addition, ionomics may also offer new hope for early detection of cancer, whose predictive power has been demonstrated for some types of cancer in recent years. Future studies should aim to improve our understanding of the relationship between metal dyshomeostasis and different types/subtypes and stages of cancer.

### 3.4. Other Complex Diseases

In recent years, ionomic studies have also been reported for several other diseases. For example, Prodanchuk et al. analyzed the concentrations of approximately 20 trace elements in the whole blood of patients with end-stage renal disease (ESRD), and found that levels of most examined elements were significantly increased in ESRD patients (especially nonessential and toxic trace elements) whereas the level of the essential element Se was significantly decreased, implying that monitoring and elimination of accumulated toxic elements as well as increasing Se uptake should be considered for ESRD patients receiving hemodiafiltration [100]. Herman et al. analyzed the concentrations of selected metals in the saliva and blood from patients with periodontitis and reported significantly increased concentrations of Cu, Mg, and Mn in the saliva of the periodontal patients, which may be helpful for the development of new diagnostic and prognostic biomarkers of periodontal disease [101]. Edvinsson et al. measured levels of a variety of trace elements in the aortic tissue and serum of patients with thoracic aortic dissection and observed somewhat similar alternation patterns of these elements in both tissues (such as decreased levels of Cu and Zn), indicating a different pathogenesis in aortic dissection than previously proposed [102]. Ilyas et al. analyzed the concentrations of trace metals in the blood and scalp hair of patients with ischemia heart disease (IHD), which revealed significantly different elemental profiles in the two tissues and suggested that metal imbalance may help to get a better understanding of the development and prevention of IHD [103]. Very recently, Shen et al. performed urinary ionomic analysis from patients undergoing cardiac surgeries and built a cardiac surgery-associated acute kidney injury (AKI) indication model based on differentially changed elements between AKI and non-AKI groups [104]. This model (named urinary ion index) is not only a novel and early indicator of cardiac surgery-associated AKI, but also associated with the 30-day survival after surgery. Future efforts are needed to investigate the ionomic network for many other complex diseases.

## 4. Conclusions and Perspectives

In the recent decade, ionomics has been rapidly developing and widely used in exploring the metabolism and homeostasis of chemical elements in different organisms such as plants and animals. However, our understanding of the relationship between different ions and the pathogenesis of various diseases is limited. This review is written to provide an overview of the application situation and the development prospect of ionomics in the study of complex disease, and to open up the possibility of using it as a new platform to help improve early diagnosis, prognosis evaluation, and therapy of these diseases. We briefly introduce major analytic methods for ionomics and, more importantly, discuss how ionomics can be applied in the study of several complex diseases such as diabetes, AD, and cancer. Disease ionomics can not only help to reveal the dynamic variation characteristics of different elements and their interactions in the pathogenesis of complex diseases, but also provides new ideas for early clinical diagnosis, risk assessment, and curative effect evaluation of related diseases. However, it should be admitted that current work about disease ionomics is just the beginning, in which the use of bioinformatics and network-based approaches is still limited. As ionomic data offer a wealth of information to be explored, it is urgent to develop new and effective algorithms for data processing and analysis. In addition, the use of ionomics has not yet been adopted for many other complex diseases so far, indicating a highly promising future for this newly developed area. With the increasingly popular use of “omics” techniques, ionomics will definitely be conducted for many other common complex diseases, which may lead to significant advances in our understanding of the dynamic network of elements and its relationship with the onset and progression of these diseases. In the future, with the availability of multi-omics data and the advent of new computational methods for high-throughput data analysis, integration of ionomic and other omic data will provide valuable information for the investigation of the roles that minerals and trace elements play in human health and disease and promote clinical translation (e.g., etiology, diagnosis, and therapy) of these achievements.

## Figures and Tables

**Table 1:** Major ionomic results for complex diseases.

Disease	Population	Number of Subjects (Disease/Control)	Tissue	Elevated Elements	Decreased Elements	Potential Confounders Adjusted or Matched	Ref.
*Diabetes*							
T2D ^1^	Moscow region, Russia	93/1236	Hair	K, Na, Hg	Ca, Mg, Zn, Co	Sex	[59]
T2D	Guanajuato, Mexico	76/12	Serum	Al, Cd, Cu, Mn, Hg, Ni	Cr, Co, V	Smoking status, other chronic diseases	[54]
			Urine	Cr, As, Cu, Zn	Cd, Co, Pb, Mn, Mo, Ni, Se		
T2D	Shanghai, China	122/854	Plasma	Cu, P, S	Mg, Cr, Se	Age, sex, BMI ^2^	[60]
T2D	Beijing and Shanghai, China	747/1368	Urine	Ni	-	Age, sex, region, residence, education, smoking and drinking status, physical activity, family history of diabetes, BMI	[61]
T2D	Suzhou, China	122/429	Plasma	V, Cr, Mn, Cu, Zn, As, Se, Sr, Pd, Cd, Cs, Ba	-	Age, sex, BMI, family history of diabetes, smoking and drinking status	[62]
T2D	Tanta, Egypt	40/36	Serum	Cu	Zn, Se, Fe, Mn, Cr, Mg, As	Age, sex, weight	[63]
T2D complication	Cartagena, Spain	31/43	Saliva	-	Co	-	[64]
			Plasma	Sr	-		
T2D complication (microvascular)	Ankara, Turkey	118/40	Blood	-	Mg, Cr	-	[65]
T2D	Nord-Trøndelag County, Norway	128/755	Blood	Cd, Cr, Fe, Ni, Ag, Zn	Br	Age, sex, BMI, waist-to-hip ratio, education, income, smoking status, family history of diabetes, seafood intake, alcohol consumption	[66]
T2D	Sardinia region, Italy	68/59	Blood	-	Cr, Mn, Ni	-	[67]
T1D ^3^	Sardinia region, Italy	192/59	Blood	-	Cr, Mn, Ni, Pb, Zn	-	[67]
T1D	Miami, U.S.	63/65	Serum	Cu, Mo	Mn, Zn, Se	Age, sex, BMI	[68]
GD ^4^	Padova, Italy	28/19	Placenta	Se	Cd	-	[69]
GD	Padova, Italy	36/36	Placenta	Hg, Si	Fe, Cu, Mn, Cd	-	[70]
		35/30	Mother blood	Cu	K, Co, Al, Rb, Sb		
		35/34	Umbilical cord blood	Ca, Na, Co, Cu, Mo, Zn, Al, Ti	Fe, K, Mn, Si, Rb		
*Neurodegenerative disease*							
AD ^5^	Göteborg, Sweden	173 (AD), 87 (AD + vasc)/54	Plasma	Mn, Hg	Co, Se, Cs	Age	[71]
			CSF	-	V, Mn, Rb, Sb, Cs, Pb		
AD	Huelva, Spain	30 (AD), 16 (MCI)/30	Serum	Al, Fe	Mn, Zn, Se	Age	[72]
AD	Kayseri, Turkey	62/60	Nail	-	Mn, Fe, Cu, Zn, Cd, Hg	Age, sex, living environment, health status	[73]
			Hair	Na, K	Al, Mn, Fe, Co, Cu, Cd, Hg, Pb		
PD ^6^	Rome, Italy	26/13	Serum	Ca, Mg	Al, Cu	Age	[74]
			Urine	Ca, Fe, Si	-		
PD	Mysore, India	52/25	Serum	K, Mg, Cu, P	Al, S, Fe, Zn	Age, sex	[75]
PD	Rome, Italy	42/20	CSF	-	Co, Cr, Fe, Pb, Si, Sn	Age, sex	[53]
PD	Wenzhou, China	238/302	Plasma	Fe, Se	Zn	Age, sex	[76]
PD	Kolkata, India	50/60	CSF	Ca, Cr, Pb, Mg	Al, Co, Fe, Mn, Si, Zn	Age, sex	[77]
		250/280	Serum	Al, Ca, Pb, Mg	Cu, Fe		
HD ^7^	Torino, Italy	18/18	Blood	As, Cr, Fe, Se, Zn	Pb, Sb, V	-	[78]
ALS ^8^	Stockholm, Sweden	17/10	CSF	Mn, Al, Cd, Co, Cu, Zn, Pb, V, U	-	-	[79]
		15/9	Plasma	Hg, Ag	-		
ALS	Milan, Italy	6/5	Blood	Mn, Al, Se	As	Age	[80]
ALS (severe vs. less severe)	Sassari, Italy	17 (severe), 16 (less severe)/30	Blood	Pb	Hg, Cu, Se	Smoking and drinking status, exposure to heavy metals	[81]
			Urine	-	Mg, Ca		
			Hair	Zn	Hg, Pb, Cu		
*Cancer*							
Prostate cancer	Shanghai, China	60/55	Hair	K, Fe, Cu, Se	Mg, P, Ca, Cr, Mn, Zn	-	[82]
Prostate cancer	Islamabad, Pakistan	74/66	Blood	Fe, Mn, Ni, Cd, Pb	Zn	Age, habitat, food habits	[83]
		67/67	Scalp hair	Fe, Mn, Ni, Cr, Cd, Pb	Zn		
		60/60	Nail	Mn, Ni, Cr, Cd	-		
Prostate cancer	Kuala Lumpur, Malaysia	50/50	Hair	Cu, Mn	Se, Zn	Age, ethnicity, family history, smoking and alcohol consumption	[84]
			Nail	Fe, Mn	Se, Zn		
Prostate cancer	Singapore	141/114	Serum	Mn, Ni, Zn, As, Se, Sb, Pb	-	Age, ethnicity, BMI, prostate-specific antigen level, eye color, skin color, family cancer history, smoking status, marital status, education	[85]
Prostate cancer	Mecca, Saudi Arabia	40/52	Serum	Cu, Fe	Se, Zn, Mn	Age	[86]
NSCLC ^9^	Kocasinan/Kayseri, Turkey	67/74	Hair	Au, Bi, Ca, Co, Cr, Cu, Ga, Hg, K, Ni, Rb, Sb, Sc, Ti, V	Ag, Cd, Fe, Zn	Age, ethnicity, geographic origin, smoking status	[87]
Lung cancer	Huelva, Spain	48/39	Serum	V, Cr, Cu	-	-	[88]
		48/39	Urine	Cd	-		
		24/31	Bronchoalveolar lavage fluid	Mn	-		
Lung cancer	Lahore, Pakistan	95/91	Serum	Cd, Cr, Pb, Hg, As, Ni	Co, Fe, Mg, Na, K, Zn, Se	Age, sex, geographical location, history of cancer, alcohol consumption	[89]
Pancreatic cancer	Madrid, Spain	118/399	Toenail	Cd, As, Pb	Se, Ni	Age, sex, region, smoking status, education, past history of diabetes	[90]
PDAC ^10^	Oxford, U.K.	21/46	Urine	Cu, Zn	Ca, Mg	Age, sex	[91]
Colorectal cancer	Vigo, Spain	38/38 (paired)	Tumor vs. non-tumor	Mn, Se, Cu, Al, Fe, Mg, Ca, K, P, S	Cd	-	[92]
Colorectal cancer	Tehran, Iran	50/50 (paired)	Tumor vs. non-tumor	Zn, Cr, Cu, Al, Pb	Sn, Fe, Mn	-	[93]
Gastric cancer	Sanandaj, Iran	35/30	Tumor vs. non-tumor	Fe, Mg, As	Cr, Cu, Ca, Ni	Sex, cancer history, smoking and alcohol consumption	[94]
ESCC ^11^	Anyang, China	100/100	Serum	Li, Sn, Ba, Tl, P, S, Bi, U, Cr, Mn, Cu, Rb, Pb,	B, Ti, Ge, As, Se, Sr, Cs, La, V, Zn, Hg, Ca, K, Mg, Na	Age, sex, region, smoking and alcohol consumption	[95]
ESCC	Quanzhou, China	30/30 (paired)	Tumor vs. non-tumor	Mn, Se, Cu, Ti, Mg, Fe, Co	Zn, Sr, Ca	-	[96]
Laryngeal cancer	Kraków, Poland	68/73	Hair/nail	Cr, Cd, Pb	Ca, Mg, Cu, Fe, Mn, Co	Age, smoking and drinking habits, diet	[97]
Breast cancer	Springfield, USA	47/84	Urine	Cu, Pb	-	Age	[98]
Gallbladder cancer	Shanghai, China	259 (gallbladder cancer), 701 (gallstones)/851	Serum	Cd, Cr, Cu, Mo, V	As, B, Ca, Fe, Li, Mg, Se, S	Age, sex, BMI, smoking and alcohol consumption, levels of triglycerides and cholesterol	[99]
*Other diseases*							
End-stage renal disease	Kyiv, Ukraine	41/61	Blood	B, Al, V, Cr, Mn, Zn, Sr, Cd, Ba, Pb	Ni, As, Se, Rb	-	[100]
Periodontal disease	Poznan, Poland	31/29	Saliva	Cu, Mg, Mn	-	Age, sex, smoking status	[101]
Thoracic aortic dissection	Uppsala, Sweden	21/23	Serum	Fe	Ca, V, Cu, Zn	Age	[102]
		18/10	Aortic tissue	Fe	Cu, Zn		
Ischemia heart disease	Islamabad, Pakistan	100/100	Blood	Cd, Co, Cr, Fe, K, Li, Mn, Na, Pb	Ca	Age, sex, habitat, smoking habits	[103]
		154/133	Scalp hair	Ca, Cd, Fe, K, Li, Pb, Sr	Na		
Acute kidney injury	Shanghai, China	92/169	Urine	Al, Fe, Co, Cu, Zn, Ag, Cd, W, Pb	Cs	Age, weight, height, BMI, history of other diseases, coronary artery bypass graft	[104]

^1^ T2D: type 2 diabetes; ^2^ BMI: body mass index; ^3^ T1D: type 1 diabetes; ^4^ GD: gestational diabetes; ^5^ AD: Alzheimer’s disease; ^6^ PD: Parkinson’s disease; ^7^ HD: Huntington’s disease; ^8^ ALS: amyotrophic lateral sclerosis; ^9^ NSCLC: non-small cell lung cancer; ^10^ PDAC: pancreatic ductal adenocarcinoma; ^11^ ESCC: esophageal squamous cell carcinoma.

## Abbreviations

ICP-MS	Inductively coupled plasma mass spectrometry
ICP-OES	Inductively coupled plasma optical emission spectroscopy
XRF	X-ray fluorescence
PiiMS	Purdue Ionomics Information Management System
ANN	Artificial neural network
CSF	Cerebrospinal fluid
AD	Alzheimer’s disease
PD	Parkinson’s disease
T1D	Type 1 diabetes
T2D	Type 2 diabetes
GD	Gestational diabetes
HD	Huntington’s disease
ALS	Amyotrophic lateral sclerosis
Aβ	Beta-amyloid
3xTg-AD	Triple-transgenic AD
PC	Prostate cancer
LC	Lung cancer
NSCLC	Non-small cell lung cancer
BALF	Bronchoalveolar lavage fluid
PaC	Pancreatic cancer
PDAC	Pancreatic ductal adenocarcinoma
CRC	Colorectal cancer
ESCC	Esophageal squamous cell carcinoma
GBC	Gallbladder cancer
ESRD	End-stage renal disease
IHD	Ischemia heart disease
AKI	Acute kidney injury
